# The prevalence of amphenicol resistance in *Escherichia coli* isolated from pigs in mainland China from 2000 to 2018: A systematic review and meta-analysis

**DOI:** 10.1371/journal.pone.0228388

**Published:** 2020-02-11

**Authors:** Zhe Du, Mengya Wang, Guanyi Cui, Xiangyang Zu, Zhanqin Zhao, Yun Xue

**Affiliations:** 1 Lab of Medical Microbiology Engineering, College of Medical Technology and Engineering, Henan University of Science and Technology, Luoyang, China; 2 College of Animal Science and Technology, Henan University of Science and Technology, Luoyang, China; 3 School Hospital, Henan University of Science and Technology, Luoyang, China; University of Mississippi Medical Center, UNITED STATES

## Abstract

**Background:**

Amphenicols have been widely used in the pig industry in China, leading to varying degrees of drug resistance.

**Methods:**

The systematic review was performed according to PRISMA (Preferred Reported Items for Systematic Reviews and Meta-Analysis) recommendations on studies investigating the prevalence of amphenicol-resistant *Escherichia coli* (*E*. *coli)* isolated from pig in mainland China from 2000 to 2018, a random-effects model was selected, then followed by meta-analysis.

**Results:**

A total of 103 articles were included in the study. The results of the meta-analysis revealed that the pooled summarized prevalence of resistance to chloramphenicol (CAP) was 72.31% (95% confidence interval (95% CI) = 67.12%–77.23%) and to florfenicol (FF) was 58.64% (95% CI = 52.48%–64.67%). During the past 18 years, the resistance rate to CAP remained high initially but then declined rapidly after 2012, whereas the resistance rate to FF plateaued (54.13%–59.60%) from 2000–2018. In different parts of China, the rate of resistance to amphenicols among *E*. *coli* isolates was fairly consistent, with the exception of the north and northwest regions.

**Conclusions:**

In 2002, the veterinary use of CAP was prohibited and its resistance levels in *E*. *coli* isolated from pigs was initially maintained at a high level but then showed an obvious downward trend in recent years. Resistance to commonly used FF remained at a high but stable level.

## Introduction

Over the past two decades, a range of antibiotics have been widely used in the pig industry in China. As a result, pig morbidity and mortality greatly reduced, and farmers achieved good economic benefits. However, at present, antibiotic resistance among clinical pathogens isolated from pig farms is reportedly increasing [1,2].

One type of antibiotics, namely amphenicol, was the first choice therapy to treat bacterial diseases of pigs in China. Amphenicols have broad-spectrum inhibitory effects on both Gram-positive and negative bacteria, but are particularly effective against Gram-negative bacteria. They include chloramphenicol (CAP) and its representative derivatives, such as thiamphenicol (TAP) and florfenicol (FF). Chloramphenicol is produced by Venezuelan streptococcus. Chloramphenicol can reversibly bind to the 50S subunit of ribosomal protein, blocking its transpeptidase activity and interfering with amino acid-tRNA terminal binding to the 50S subunit, thereby inhibiting the formation of new peptide chains and inhibiting the protein synthesis of bacteria [3,4]. It was once widely used in China, but has been forbidden for use in food-producing animals since 2002, as the common side effects include bone marrow suppression, aplastic anemia, nausea and diarrhea in humans [5–7]. Thiamphenicol is a CAP derivative (second generation), which possesses a -CH_3_SO_2_ group instead of a -NO_2_ group. Thiamphenicol has a similar antibacterial mechanism to CAP, but lower toxicity than CAP. It does not produce aplastic anemia, but can induce reversible myelosuppression and has strong immunosuppressive effects [8,9]. Florfenicol is a monofluoride derivative of TAP (third generation), in which the -OH is replaced with -F. Compared with the previous two generations, FF is a low toxicity, high efficiency, relatively safe veterinary drug. In addition, the fluorine atom in the FF molecule replaces the hydroxyl groups on the propane chain in both CAP and TAP. This structure means that the antibiotic-derived acetyltransferase cannot produce resistance to FF. Therefore, many CAP and TAP-resistant bacteria have retained sensitivity to FF, and it has become one of the most commonly used veterinary antibiotics over the last decade in China [10,11].

*Escherichia coli* can cause piglet yellow dysentery, white diarrhea, edema disease, weaning diarrhea and other diseases in pigs. *Escherichia coli* can also co-infect pigs along with other bacteria or viruses, leading to increased morbidity and mortality in these animals. Some evidence has suggested that *E*. *coli* has become increasingly resistant to amphenicols, such as CAP [1,12]. Mutant strains of *E*. *coli* can produce enzymes that degrade or inhibit the activity of the antimicrobial agents, modify the antibiotic target sites, reduce the permeability of the membrane to the drug or alter the metabolic pathway or metabolic state, thereby gaining resistance to CAP [13]. *Escherichia coli* can also acquire resistance genes from other microorganisms or environments, such as CAP-resistant *cmlA* gene cassettes and R plasmids [14,15]. In previous reports from the 1990s, *E*. *coli* isolates from pigs showed varying degrees of resistance to commonly used veterinary antibiotics in China, such as CAP, aminoglycosides and sulfonamides [1,16].

By reviewing past reports, it became clear that *E*. *coli* resistance to amphenicols may show patterns with respect to different regions and time periods. There is also a lack of studies presenting a systematic comparison and analysis of drug resistance among *E*. *coli* in China. Therefore, we searched both Chinese and English databases for all articles describing amphenicol resistance among *E*. *coli* isolated from pigs in China from 2000 to 2018 and conducted a meta-analysis.

## Materials and methods

### Search strategy and selection criteria

The PubMed, EMBASE, EBSCO, ISI Web of Knowledge, Chinese National Knowledge Infrastructure (CNKI), and Wanfang (Chinese) databases were searched (between January 2000 and December 2018) by using the following keywords: “pig” or “swine” and “drug resistant” or “antibiotic resistant” or “antimicrobial resistance” or “amphenicols” or “chloramphenicol” or “thiamphenicol” or “florfenicol” and “*Escherichia coli*” or “*E*. *coli*” and “China”. In the Chinese databases, the keyword “China” was removed from the search words because some reports in Chinese presented data from provinces or regions in China but did not mention the word “China” in the report. Then, following the PRISMA (Preferred Reported Items for Systematic Reviews and Meta-Analyses) statement [17], titles and abstracts of the articles were initially screened by two authors, with a third researcher consulted in cases of disagreement. Full—text articles, excluding reviews, conference abstracts and book chapters, were retrieved. All articles reporting amphenicol resistance in *E*. *coli* isolated from pigs in mainland China were considered when presenting a resistance categorisation with not less than 10 minimum number of samples being considered. Additional articles were identified from the reference lists and review articles.

### Inclusion and exclusion criteria

The inclusion criterion was studies in which the drug resistance rates and the total number of *E*. *coli* strains were available (or data were available to calculate these values).

The exclusion criteria consisted of: 1) studies with duplicate data or insufficient data; 2) data from reviews and abstracts; 3) *E*. *coli* strains isolated out of the study range (from January 2000 to December 2018); 4) *E*. *coli* strains isolated from other countries other than China; 5) *E*. *coli* strains that were not selected randomly. Before drug resistance tests, *E*. *coli* strains were pre-selected by other standards, such as containing certain genes; 6) *E*. *coli* strains isolated from other animals (i.e., not pigs); 7) data from treated foods (i.e., not pork) or pig farm environments (i.e., not water); 8) *E*. *coli* strains isolated from fewer than 10 cases. The references of the selected articles and reviews were also scanned manually to identify any additional eligible studies.

### Statistical analysis

Data on the total prevalence of resistance for amphenicols in different regions and years were calculated independently using Microsoft Excel 12.0 (Microsoft Co., Ltd., Washington, USA). All data manipulation and statistical analyses were performed using R-3.4.4 in this meta-analysis. The raw proportions were transformed using the Freeman-Tukey double arcsine transformation to normalize the sampling distribution of the proportions and to stabilize their variances [18]. A random-effects model (Der Simonian and Laird method) was selected, given the possibility of significant heterogeneity between the studies [19]. Heterogeneity among the included studies was assessed via I^2^ tests. Values close to 0% indicate no heterogeneity, whilst values close to 25%, 50% and 75% correspond to low, moderate and high heterogeneity, respectively [20]. The data were entered and a statistical analysis was performed and presented as a forest plot. Forest plots were generated to show the prevalence proportions with corresponding 95% CIs for each study and the overall random-effects pooled estimate. To evaluate the impact of publication bias, funnel plots were obtained plotting the Freeman—Tukey double arcsine event rate against the corresponding standard error, with the studies symmetrically distributed in the absence of publication bias. To avoid misinterpretation of the funnel plots, Egger’s regression test was also employed [21]. The results were considered to have no publication bias when P > 0.05.

## Results

### Search results

**Fig 1:**
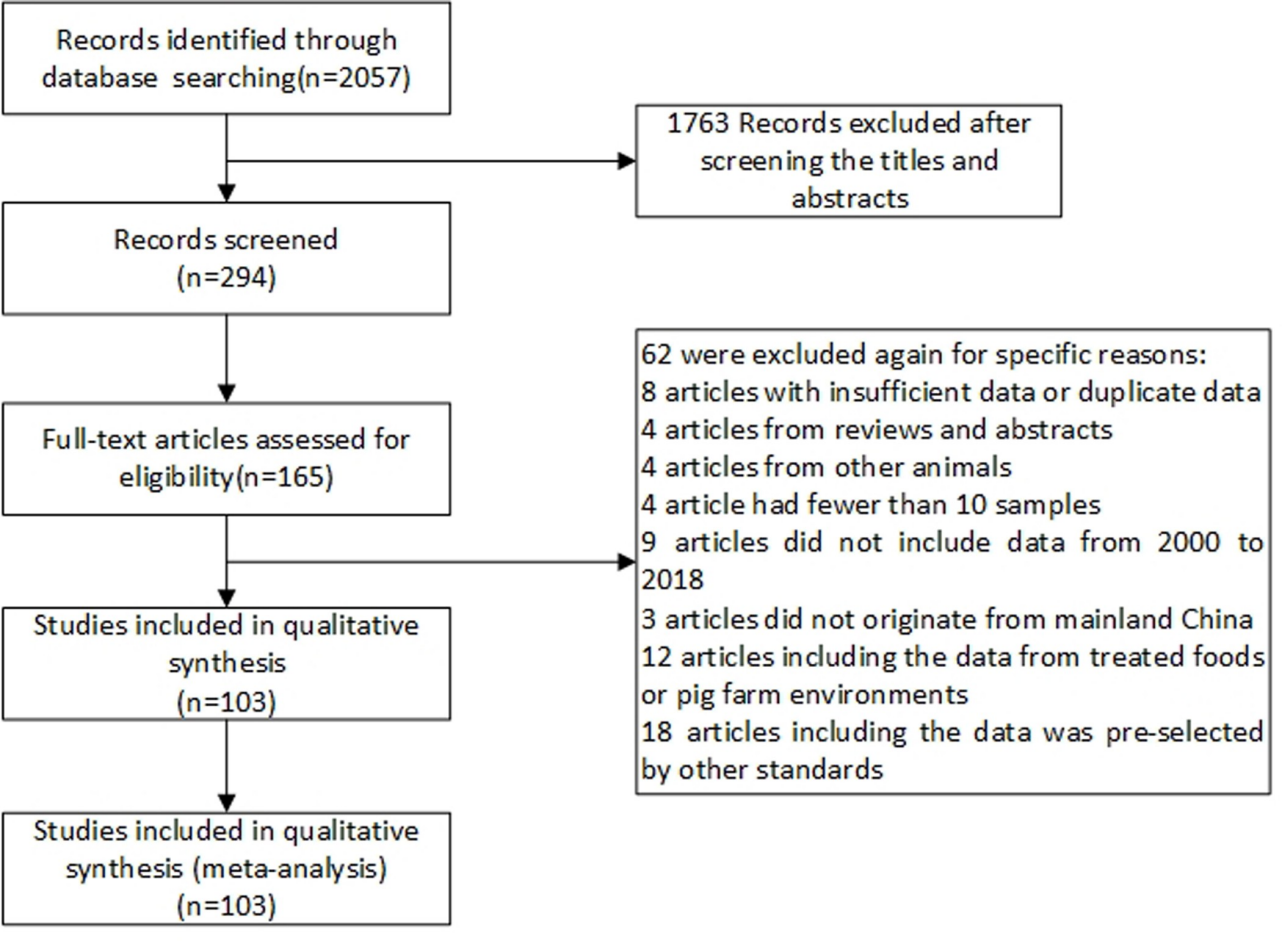
PRISMA flow diagram. Flow chart depicting the study selection process.

In accordance with the literature retrieval method referred to in the “Materials and methods”, we identified 2,057 articles published from 2000 to 2018. After an initial evaluation of the titles and abstracts, 1,763 articles were excluded due to their irrelevance or duplication. The full text of the remaining articles (294) was reviewed. After excluding those described only the prevalence of *E*. *coli* and were not able to extract information regarding the rates of resistance, and those provided rates of resistance but did not describe specific drugs, at final, a total of 165 articles were included. Among the 165 articles, 62 were further excluded for specific reasons: eight articles with duplicate or insufficient data, four articles from reviews and abstracts, nine articles did not include data from 2000 to 2018, three articles did not originate from mainland China, 18 articles included data pre-selected by other standards, four articles concerned other animals, 12 articles included data from treated foods or pig farm environments, and four articles had fewer than 10 samples. Finally, 103 articles were included in this systematic review and meta-analysis. The screening flow is shown in Fig 1.

### Characteristics of the eligible studies

S1 Table is the PRISMA checklist and S2 Table shows the characteristics of the final 103 studies eligible for inclusion. These studies were grouped based on geographical region in which CAP/FF resistance was detected: eastern (n = 14/13), southern (n = 7/9), northern (n = 4/5), northwestern (n = 3/2), southwestern (n = 8/14), northeastern (n = 9/7) and central China (n = 8/10). In addition, according to the Social Development of China, we divided the enrollment time into three groups: 2000–2006, 2007–2012 and 2013–2018. For these three periods, the number of isolates resistant to CAP/FF was highest in 2007–2012 (2582/5304), followed by 2013–2018 (1547/2164) and 2000–2006 (1257/731). For one study, which included 133 samples with TAP resistance, the drug resistance rate was not available.

#### Overall prevalence of drug resistance in mainland China

Our systematic review and meta-analysis based on 57 studies with 7,392 samples demonstrated that the pooled summarized prevalence of CAP was 72.31% (95% CI = 67.12%–77.23%) (S1 Fig), with a high level of heterogeneity between the estimated rates (I^2^ = 95.4%, p<0.0001) (Table 1). In a total of 64 studies, 10,038 samples were reported with FF resistance. Table 1 also shows the rate of resistance to FF (58.64%; 95% CI = 52.48%–64.67%) (S2 Fig.), with a high level of heterogeneity between the estimated rates (I^2^ = 97.3%, p = 0).

**Table 1:** The overall prevalence of the Chloramphenicol/ Florfenicol resistance in mainland China.

Drug	Total	Prevalence of Drug Resistance(95% CI)	n/N	No. of Studies	Heterogeneity Test	
		(%)			I^2^(%)	P
CAP	NA	72.31 (67.12–77.23)	5222/7392	57	95.4	< 0.0001
FF	NA	58.64 (52.48–64.67)	5802/10038	64	97.3	= 0

NOTE. n: number of events; N: total number of samples from the studies. NA: the data were not applied to the statistical calculation. CAP: chloramphenicol. FF: florfenicol.

#### The prevalence of drug resistance in different regions of China

**Fig 2:**
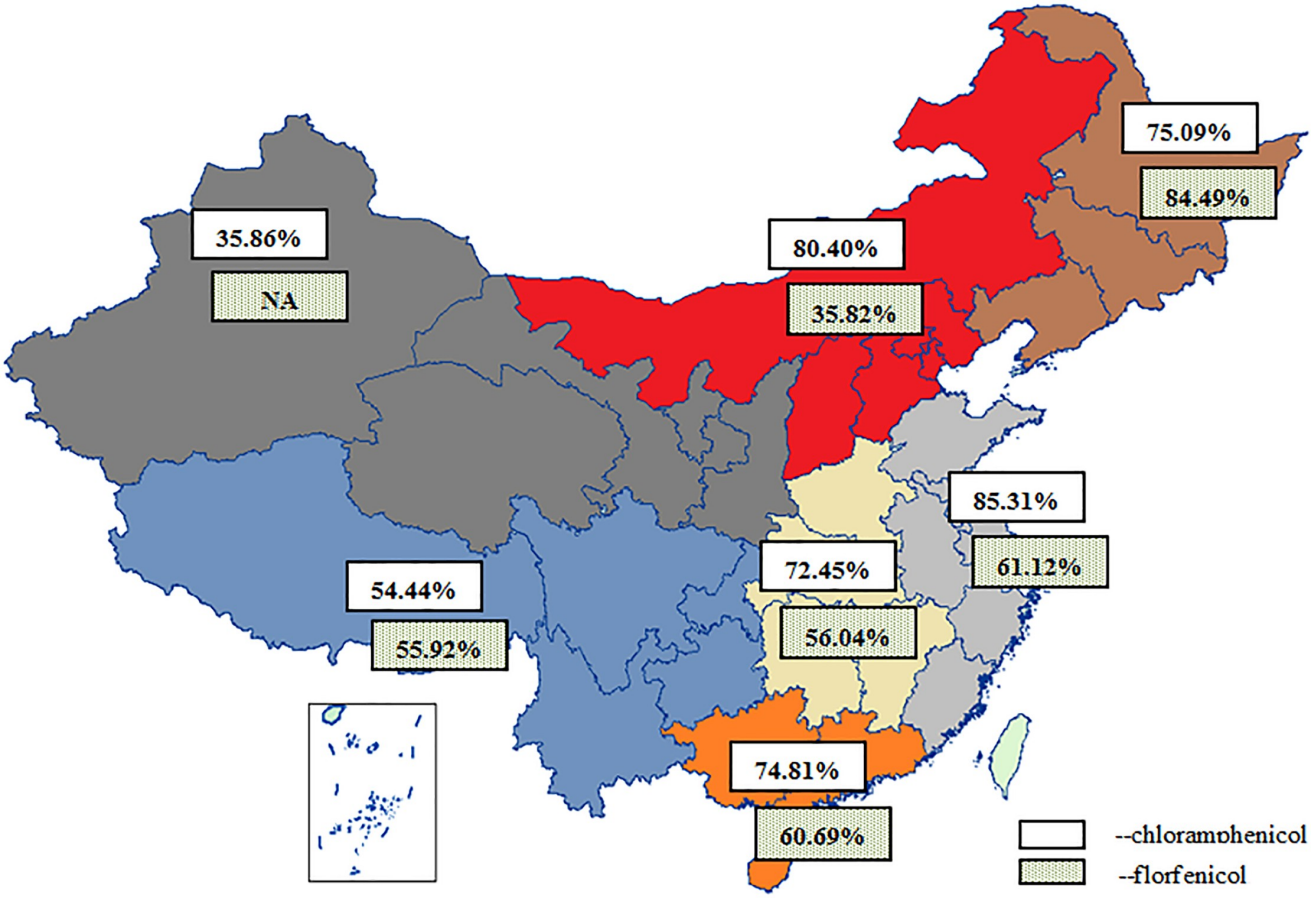
Drug-resistant status in the different regions of China.

Fig 2 is a map of the prevalence of CAP and FF in the different regions of China over the time period 2000–2018. The map clearly shows that the regional pooled prevalence of CAP resistance in northwestern China was 35.86%, lower than that in other regions of China (e.g., southern, eastern and central China), and north China was the area with the lowest prevalence of FF resistance (35.82%). Moreover, the regions with a prevalence of CAP resistance higher than that of FF resistance included eastern, southern, central and northern China, particularly northern China (80.40% vs. 35.82%). Comparatively, the prevalence of CAP resistance was lower than that of FF resistance in northeastern China (75.09% vs. 84.49%). The corresponding 95% CIs are shown in Table 2.

**Table 2:** The prevalence of the Chloramphenicol/Florfenicol resistance in the different regions.

Areas	Drug	Prevalence of Drug Resistance(95% CI)	n/N	No. of Studies	Heterogeneity Test	
		(%)			I^2^(%)	P
Central China	CAP	72.45(60.83–86.29)	360/497	8	93.0	< 0.0001
	FF	56.04(45.48–69.06)	454/801	10	94.6	< 0.0001
Eastern China	CAP	85.31(77.50–91.69)	941/1144	14	91.1	< 0.0001
	FF	61.12(53.42–68.30)	826/1433	13	86.2	< 0.0001
Southern China	CAP	74.81(58.91–86.03)	1136/1502	7	95.6	< 0.0001
	FF	60.69(49.22–71.09)	1415/2144	9	94.2	< 0.0001
Northern China	CAP	80.40(54.52–93.35)	180/214	4	85.5	< 0.0001
	FF	35.82(16.92–75.81)	97/267	5	95.8	< 0.0001
Northwestern China	CAP	35.86(20.64–51.09)	130/301	3	84.5	0.0016
	FF	NA	NA	NA	NA	NA
Southwestern China	CAP	54.44(41.12–67.15)	529/943	8	92.2	< 0.0001
	FF	55.92(38.58–73.25)	1018/1769	14	98.9	< 0.0001
Northeastern China	CAP	75.09(59.64–87.90)	909/1299	9	96.8	< 0.0001
	FF	84.49(72.62–93.58)	1125/1244	7	94.9	< 0.0001

NOTE. n: number of events; N: total number of samples from the studies. NA: the data were not applied to the statistical calculation. CAP: chloramphenicol. FF: florfenicol.

#### The prevalence of drug resistance over different time periods in China

As shown in Table 3, the prevalence of CAP resistance exhibited an initial increasing trend, then decreased during 2000–2018, with the highest resistance rate (80.57%) in 2007–2012 and the lowest rate (47.86%) in 2013–2018. However, the rate of resistance to FF has plateaued (55%–60%) over the last two decades.

**Table 3:** The prevalence of the Chloramphenicol/Florfenicol resistance during the different periods.

Periods	Drug	Prevalence of Drug Resistance(95% CI)	n/N	No. of Studies	Heterogeneity Test	
		(%)			I^2^(%)	P
2000–2006	CAP	65.99(55.73–75.58)	799/1257	14	91.9	< 0.0001
	FF	54.13(47.45–60.74)	397/731	8	66.8	0.0036
2007–2012	CAP	80.57(71.36–88.41)	1957/2582	17	96.3	< 0.0001
	FF	59.60(50.54–68.35)	2936/5304	25	97.6	< 0.0001
2013–2018	CAP	47.86(32.00–64.15)	902/1547	8	96.4	< 0.0001
	FF	57.01(44.54–69.05)	1209/2164	19	96.4	< 0.0001

NOTE. n: number of events; N: total number of samples from the studies. NA: the data were not applied to the statistical calculation. CAP: chloramphenicol. FF: florfenicol.

### Publication bias

**Fig 3:**
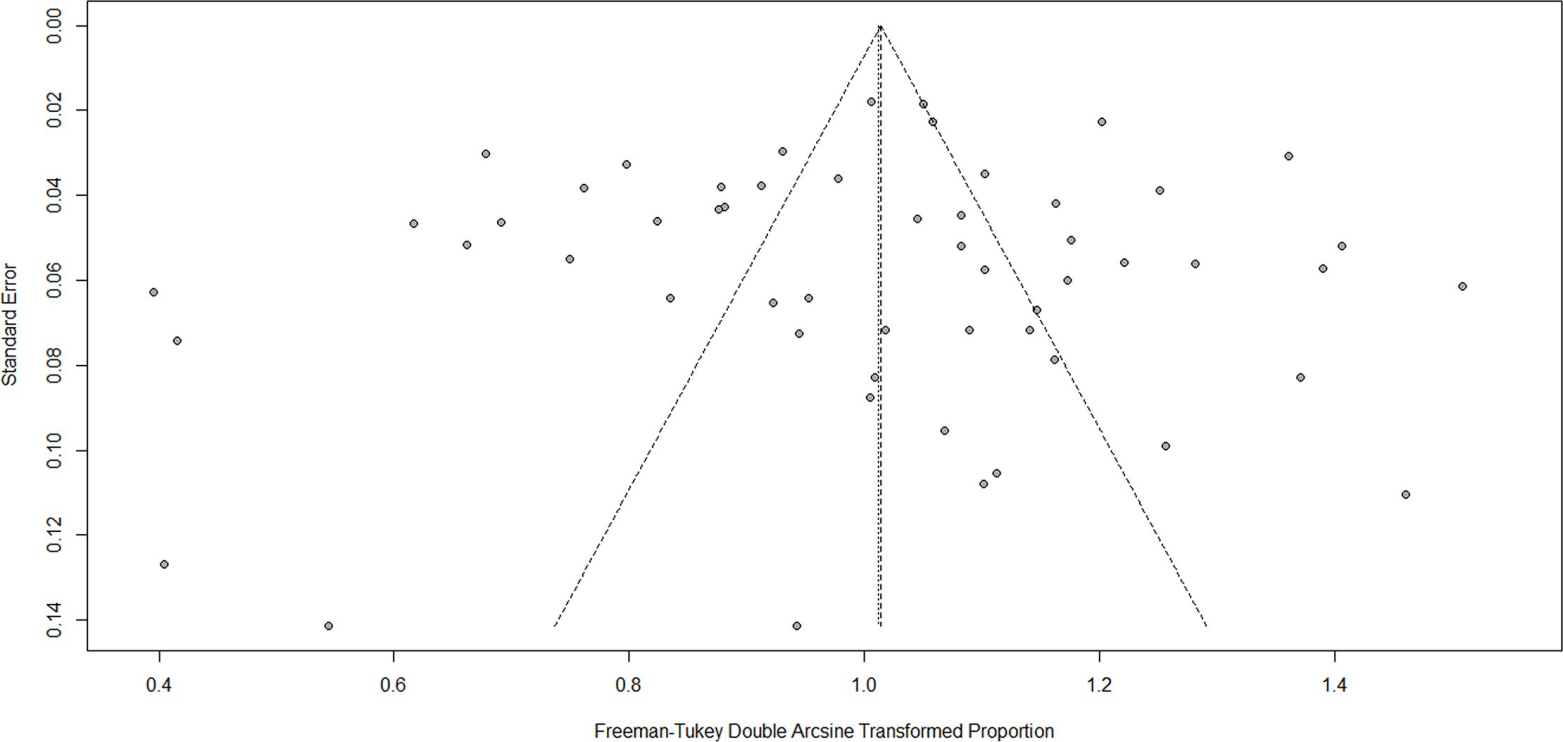
Funnel plot (CAP).

**Fig 4:**
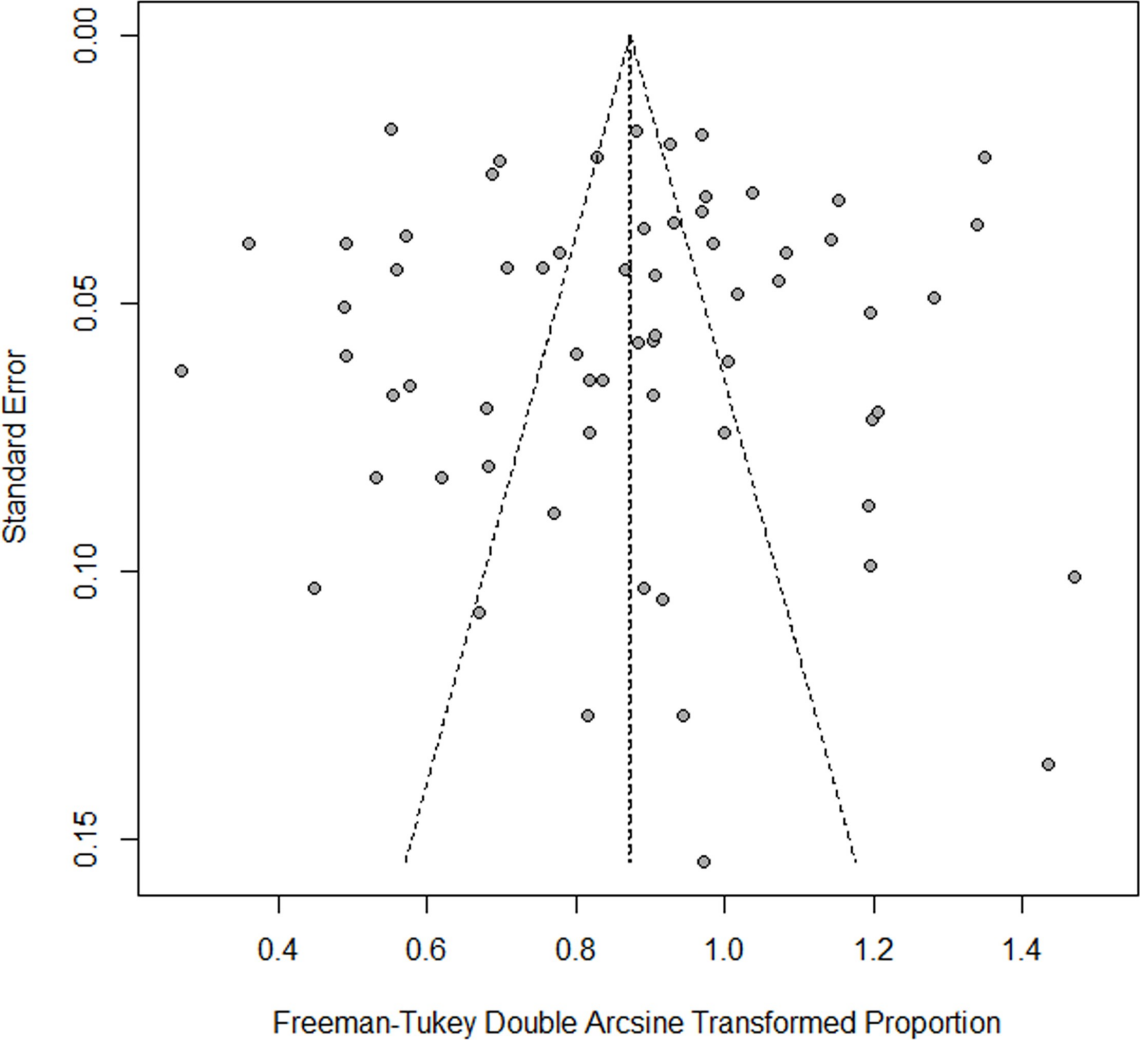
Funnel plot (FF).

The funnel plots showed no publication bias for CAP and FF (see Figs 3 and 4), which was also confirmed from Egger′s test (CAP, p = 0.8221; FF, p = 0.9481).

## Discussion

From early in this century, amphenicols were widely used in the pig industry and *E*. *coli* resistance to these antimicrobials was monitored in China. However, the data were sporadic, most published in Chinese and did not draw a lot of attention worldwide. In this study, a meta-analysis was performed to understand recent trends in amphenicol resistance in *E*. *coli* isolated from pigs in China. In this systematic review and meta-analysis, we found that amphenicol resistance is ubiquitous in *E*. *coli* isolated from pigs, with pooled summary estimates for the prevalence of CAP resistance of 72.31% and the prevalence of FF resistance of 58.64%.

According to our data, during the past 18 years (2000–2018), the resistance rate to CAP initially remained high, then declined rapidly after 2012. CAP has been forbidden for use in pigs since 2002 in China, because the drug residues are accompanied by potential health risks in humans [7]. However, *E*. *coli* resistance to CAP continued to increase from 2000–2006 (65.99%) and from 2007–2012 (80.57%) after the ban. There may be a few possible explanations for this. First, there is some evidence that CAP was still used illegally by some individuals in China during this time. Second, without the use of CAP, CAP-resistant genes still could be transferred from other bacteria in the environment into pigs. Third, CAP resistance may be co-acquired along with other types of antibiotic resistance. It has been reported that co-resistance can develop because the use of dihydrostreptomycin and trimethoprim in pigs apparently contributes to the selection of CAP-resistant strains of *E*. *coli* [22]. Thus, CAP resistance persistently increased under a high level of antibiotic pressure in the environment. Fortunately, there has been an obvious downward trend in CAP resistance since 2012, with a rate of 80.57% for 2007–2012 compared with 47.86% for 2013–2018. This might be due to the stricter regulation of drugs in China's health sector in recent years. From 2016 to date, no eligible article was found on the CAP resistance of *E*. *coli* isolates from pigs in China. It was also evident that veterinarians no longer used CAP in China. Therefore, the CAP resistance rate among *E*. *coli* isolates decreased overall.

Today in China, FF is the only commonly used amphenicol and is among the top three antibiotics currently used to prevent pig disease [23]. However, the level of resistance to FF increased to a point that its effects became limited only several years after it was approved for use in China in 1999 [24]. In our results, the prevalence of FF resistance was high but generally remained stable over the 2000–2018 time period analysed, i.e., pooled rates were 54.13% (2000–2006), 59.60% (2007–2012) and 57.01% (2013–2018). Thiamphenicol is often used in fish but seldom in pigs, as it is less effective and more toxic compared with FF in curing pig diseases [25]. That is why only one eligible article on TAP was found in this study. These results illustrated the serious situation with regard to amphenicol resistance among *E*. *coli*, and the need for close monitoring.

The serious amphenicol resistance levels in China may be attributable to the following reasons. China is the world's largest pig breeding country. Over the past two decades, the large-scale farming model has been increasingly adopted in mainland China, gradually replacing individual small-scale farming. Farming has become standardized, centralized and large-scale in the pig industry. With this change in farming patterns, the prevalence of pig infectious diseases has increased, as has the complexity of the causative agents. At the same time, veterinary antibiotics have been widely applied in the pig industry, or even abused at times. Studies have indicated that, at a national level, the extent of use of specific antimicrobials strongly correlates with the level of resistance towards these agents in commensal *E*. *coli* isolates in pigs [26]. Taken together, these events in China may have contributed to selecting pressure of *E*. *coli* and increase the emergence of amphenicol resistance of *E*. *coli*. After CAP use was prohibited in China, its resistance rate showed a downward trend, which was further evidence of the link between antibiotic use and resistance.

Over the last two decades, a high prevalence of amphenicol resistance was observed across all regions of China, but varied between different pig farms (from 35% to 80%). With the exception of the western region, large-scale farms are distributed across all parts of China, and the introduction of pig farms is relatively frequent. This inevitably increases the spread of drug-resistant pathogens among pigs nationwide. Our META analysis results indicated that the amphenicol low resistance rate areas were mostly the small, remote, mountainous areas, such as those found in northwestern regions. Those areas with the highest rates of resistance to amphenicols were mostly the large, well-stocked areas, especially in northern and eastern China.

Compared with many other countries, the prevalence of amphenicol resistance observed in China seems to be among the highest reported worldwide. For example, the overall CAP resistance rate determined in this study was 72.31%, which is much higher than some European countries (range 0%–30%) [13,26,27], but similar to other Asian countries, such as Thailand (95.9%) [28] and Korea (60.4%) [12]. Besides CAP, high levels of resistance to FF also observed. Resistance rates to FF in this study was 58.64%, which was similar to those reported for Italy (FF, 64.3%) [29]. However, these data on amphenicol resistance in other counties are sporadic and are unable to be directly compared with the results of our meta-analysis. These amphenicol resistance rate differences between countries are also likely affected by climate and antibiotic control measures implemented in particular countries.

Our meta-analysis has several limitations. Significant heterogeneity was observed among certain studies. This may be due to inconsistencies among the subject demographic information, standards of quality, and methodologies adopted by different studies. Especially, in different regions, there is difference among the climate, the measures of antibiotic use and habits for raising pigs. Those are certainly influence the results of this meta-analysis. Moreover, some of the original information from the geographic and time segment subgroup analysis were insufficient, and there were variations in the quality of the selected articles. Hence heterogeneity may be influenced by uncertain data which unfortunately prevents us from obtaining further in-depth findings.

## Conclusion

We performed a systematic review and meta-analysis on the prevalence of amphenicols-resistant *E*. *coli* isolated from pigs in mainland China from 2000 to 2018. Chloramphenicol have been prohibited from use on veterinarians in China since 2002, its resistance in *E*. *coli* isolated from pigs in China once maintained at a high level but showed an obvious downward trend in recent years. Resistance to commonly used FF remained at a high but stable level and still need to be closely monitored.

## Supporting information

S1 Table PRISMA checklist. (DOC)

S2 Table Characteristics of the eligible studies. (DOCX)

S1 Fig Forest plot (CAP). (TIF)

S2 Fig Forest plot (FF). (TIF)
