# Supplementary material for: The prevalence of amphenicol resistance in Escherichia coli isolated from pigs in mainland China from 2000 to 2018: A systematic review and meta-analysis
Source: PLoS One. 2020 Feb 11;15(2):e0228388. doi: 10.1371/journal.pone.0228388 (PMC7012437; doi:10.1371/journal.pone.0228388)
Supplement: S2 Table — (DOCX) [file pone.0228388.s002.docx]

| **No** | **First author, year** | **Time period** | **Region** | **Total case** | **Case Of Chloramphenicol(CAP) Resistance** | **Case Of Florfenicol (FF) Resistance** | **Case Of Thiamphenicol(TAP) Resistance** |
| --- | --- | --- | --- | --- | --- | --- | --- |
|  |  |  |  |  |  |  |  |
|  |  |  |  |  |  |  |  |
|  |  |  |  |  |  |  |  |
| 1 | **Lai et al.,2011** | 2007-2012 | southwestern China | 286 | 184 | 212 | NA |
| 2 | **He et al.,2013** | 2007-2012 | southern China | 720 | 542 | 490 | NA |
| 3 | **Guo et al.,2014** | 2007-2012 | southern China | 264 | 253 | 221 | NA |
| 4 | **Xia et al.,2010** | 2007-2012 | eastern China | 76 | 74 | 47 | NA |
| 5 | **Du et al.,2008** | 2007-2012 | central China | 92 | 90 | 80 | NA |
| 6 | **Xun et al.,2011** | 2007-2012 | central China | 58 | 37 | 17 | NA |
| 7 | **Zhu et al.,2015** | 2013-2018 | southwestern China | 114 | 38 | NA | NA |
| 8 | **Wang et al.,2015a** | NA | northern China | 97 | 83 | 21 | NA |
| 9 | **Li et al.,2012** | 2007-2012 | northern China | 69 | 59 | 15 | NA |
| 10 | **Ma et al.,2009** | 2007-2012 | eastern China | 193 | 133 | 117 | NA |
| 11 | **Wei et al.,2011** | 2007-2012 | southwestern China | 232 | 119 | 158 | NA |
| 12 | **Xie et al.,2011** | 2007-2012 | southern China | 40 | 34 | NA | NA |
| 13 | **Wang et al.,2015b** | 2013-2018 | southern China | 63 | 9 | 4 | NA |
| 14 | **Zhang et al.,2009** | 2007-2012 | southwestern China | 124 | 97 | NA | NA |
| 15 | **Wang et al.,2014** | 2013-2018 | northeastern China | 275 | 108 | NA | NA |
| 16 | **Liu et al.,2012** | 2007-2012 | northern China | 12 | 3 | NA | NA |
| 17 | **Yao et al.,2011** | 2007-2012 | northeastern China | 55 | 46 | 34 | NA |
| 18 | **Jiang et al.,2009** | 2007-2012 | eastern China | 66 | 66 | NA | NA |
| 19 | **Zhao et al.,2011** | 2007-2012 | northern China | 36 | 35 | 9 | NA |
| 20 | **Chen et al.,2013** | NA | eastern China | 25 | 23 | NA | NA |
| 21 | **Wang et al.,2013b** | NA | northeastern China | 48 | 35 | 42 | NA |
| 22 | **Zhan et al.,2014** | 2013-2018 | eastern China | 120 | 90 | NA | NA |
| 23 | **Xu et al.,2015** | NA | northeastern China | 488 | 425 | 465 | NA |
| 24 | **Di et al.,2008** | NA | eastern China | 47 | 31 | NA | NA |
| 25 | **Li et al.,2017** | 2013-2018 | northeastern China | 136 | 81 | NA | NA |
| 26 | **Han et al.,2008** | 2007-2012 | eastern China | 166 | 150 | NA | NA |
| 27 | **Guo et al.,2015** | 2013-2018 | southwestern China | 45 | 7 | 32 | NA |
| 28 | **Ding et al.,2008** | NA | eastern China | 48 | 40 | NA | NA |
| 29 | **Meng et al.,2014** | 2007-2012 | NA | 93 | 35 | NA | NA |
| 30 | **Huang et al.,2017** | NA | southern China | 40 | 34 | NA | NA |
| 31 | **Jiang et al.,2004** | 2000-2006 | eastern China | 21 | 17 | NA | NA |
| 32 | **Cui et al.,2005** | 2000-2006 | northeastern China | 20 | 20 | NA | NA |
| 33 | **Lei et al .,2004** | 2000-2006 | NA | 174 | 109 | NA | NA |
| 34 | **Li et al.,2006** | 2000-2006 | southwestern China | 48 | 38 | NA | NA |
| 35 | **Du et al.,2007** | 2000-2006 | northwestern China | 170 | 81 | NA | NA |
| 36 | **Du et al.,2006** | 2000-2006 | northwestern China | 15 | 2 | NA | NA |
| 37 | **Chen et al.,2007** | 2000-2006 | southwestern China | 12 | 8 | NA | NA |
| 38 | **Yu et al.,2005** | 2000-2006 | eastern China | 142 | 120 | NA | NA |
| 39 | **Tan et al.,2006** | 2000-2006 | central China | 117 | 63 | NA | NA |
| 40 | **Dai et al.,2007** | 2000-2006 | northwestern China | 116 | 47 | NA | NA |
| 41 | **Jiang et al.,2011** | 2000-2006 | southern China | 203 | 162 | 131 | NA |
| 42 | **Ji et al.,2007** | 2000-2006 | central China | 27 | 21 | NA | NA |
| 43 | **Shen et al.,2007** | 2000-2006 | northeastern China | 132 | 78 | NA | NA |
| 44 | **Qin et al.,2008** | 2000-2006 | eastern China | 60 | 33 | NA | NA |
| 45 | **Wu et al.,2018** | 2013-2018 | eastern China | 22 | 18 | NA | NA |
| 46 | **Jin et al., 2016** | 2013-2018 | NA | 772 | 551 | NA | NA |
| 47 | **Zhang et al.,2010** | NA | NA | 92 | 72 | NA | NA |
| 48 | **Xiao et al.,2008** | NA | southwestern China | 82 | 38 | NA | NA |
| 49 | **Zhao et al.,2008** | NA | central China | 75 | 60 | NA | NA |
| 50 | **Xue et al.,2010** | NA | northeastern China | 80 | 71 | NA | NA |
| 51 | **Zhou et al.,2009** | NA | central China | 36 | 26 | 12 | NA |
| 52 | **Wang et al.,2010** | NA | southern China | 172 | 102 | NA | NA |
| 53 | **Yang et al.,2002** | NA | central China | 60 | 40 | NA | NA |
| 54 | **Tang et al.,2007** | NA | NA | 480 | 365 | 260 | NA |
| 55 | **Li et al.,2009** | NA | central China | 32 | 23 | NA | NA |
| 56 | **Chen et al.,2013** | NA | eastern China | 25 | 23 | NA | NA |
| 57 | **Tang et al.,2010** | NA | eastern China | 79 | 73 | 49 | NA |
| 58 | **Wang et al.,2013a** | 2007-2012 | NA | 797 | NA | 219 | NA |
| 59 | **Shu et al.,2014** | 2007-2012 | southwestern China | 273 | NA | 187 | NA |
| 60 | **Xie et al.,2010** | 2007-2012 | southern China | 23 | NA | 4 | NA |
| 61 | **Cao et al.,2016** | 2013-2018 | southwestern China | 164 | NA | 36 | NA |
| 62 | **Ning et al.,2016** | 2013-2018 | eastern China | 164 | NA | 114 | NA |
| 63 | **Shu et al.,2012** | 2007-2012 | southwestern China | 70 | NA | 36 | NA |
| 64 | **Wen et al.,2015** | 2013-2018 | southwestern China | 38 | NA | 15 | NA |
| 65 | **Rao et al.,2014** | 2007-2012 | southern China | 606 | NA | 388 | NA |
| 66 | **Tang et al.,2010** | 2007-2012 | central China | 25 | NA | 22 | NA |
| 67 | **Xia et al.,2012** | 2007-2012 | northwestern China | 454 | NA | 187 | NA |
| 68 | **Qu et al.,2010** | 2007-2012 | eastern China | 119 | NA | 92 | NA |
| 69 | **Wen et al.,2014** | 2013-2018 | southwestern China | 13 | NA | 13 | NA |
| 70 | **Zhang et al.,2012** | NA | eastern China | 124 | NA | 77 | NA |
| 71 | **Guo et al.,2011** | 2007-2012 | eastern China | 371 | NA | 149 | NA |
| 72 | **Zhao et al.,2015** | NA | southwestern China | 198 | NA | 188 | NA |
| 73 | **Chen et al.,2015** | 2013-2018 | eastern China | 129 | NA | 75 | NA |
| 74 | **Feng et al.,2017** | 2013-2018 | southern China | 103 | NA | 95 | NA |
| 75 | **Mao et al.,2014** | 2013-2018 | southwestern China | 21 | NA | 8 | NA |
| 76 | **Pan et al.,2014** | 2013-2018 | southern China | 10 | NA | 7 | NA |
| 77 | **Gu et al.,2017** | 2013-2018 | southwestern China | 165 | NA | 20 | NA |
| 78 | **Wang et al.,2016** | 2013-2018 | northeastern China | 106 | NA | 77 | NA |
| 79 | **Li et al.,2014** | 2007-2012 | southwestern China | 129 | NA | 36 | NA |
| 80 | **Li et al.,2017a** | 2013-2018 | central China | 31 | NA | 15 | NA |
| 81 | **Ai et al.,2017** | 2013-2018 | central China | 171 | NA | 142 | NA |
| 82 | **Li et al.,2017b** | NA | northern China | 15 | NA | 8 | NA |
| 83 | **Jiang et al.,2004** | 2000-2006 | eastern China | 23 | NA | 14 | NA |
| 84 | **Zhang et al.,2005** | 2000-2006 | NA | 60 | NA | 33 | NA |
| 85 | **Yang et al.,2007** | 2000-2006 | southwestern China | 75 | NA | 45 | NA |
| 86 | **Lin et al.,2009** | 2000-2006 | NA | 133 | NA | 56 | NA |
| 87 | **Fan et al.,2008** | 2000-2006 | central China | 45 | NA | 24 | NA |
| 89 | **Li et al.,2008** | 2000-2006 | central China | 132 | NA | 62 | NA |
| 90 | **Jiang et al.,2008** | 2000-2006 | southwestern China | 60 | NA | 32 | NA |
| 91 | **Zhang et al.,2013** | 2007-2012 | northeastern China | 24 | NA | 24 | NA |
| 92 | **Gu et al.,2012** | 2007-2012 | northern China | 50 | NA | 44 | NA |
| 93 | **Wu et al.,2014** | 2013-2018 | eastern China | 51 | NA | 20 | NA |
| 94 | **Liu et al.,2015** | 2013-2018 | eastern China | 15 | NA | 10 | NA |
| 95 | **Wu et al.,2018a** | 2013-2018 | eastern China | 22 | NA | 14 | NA |
| 96 | **Wu et al.,2018b** | 2013-2018 | central China | 32 | NA | 28 | NA |
| 97 | **Tong et al.,2018** | 2013-2018 | NA | 152 | NA | 119 | NA |
| 98 | **Jin et al.,2016** | 2013-2018 | NA | 772 | NA | 460 | NA |
| 99 | **Jiao et al.,2015** | NA | eastern China | 67 | NA | 48 | NA |
| 100 | **Han et al.,2014** | NA | northwestern China | 55 | NA | 15 | NA |
| 101 | **Wang et al.,2010** | NA | southern China | 152 | NA | 75 | NA |
| 102 | **Yu et al.,2011** | 2007-2012 | central China | 179 | NA | 52 | NA |
| 103 | **Lin et al.,2009** | 2000-2006 | NA | 133 | NA | NA | 115 |

NA: Not Available.
