# Supplementary figures and images for: The prevalence of amphenicol resistance in Escherichia coli isolated from pigs in mainland China from 2000 to 2018: A systematic review and meta-analysis
Source: PLoS One. 2020 Feb 11;15(2):e0228388. doi: 10.1371/journal.pone.0228388 (PMC7012437; doi:10.1371/journal.pone.0228388)

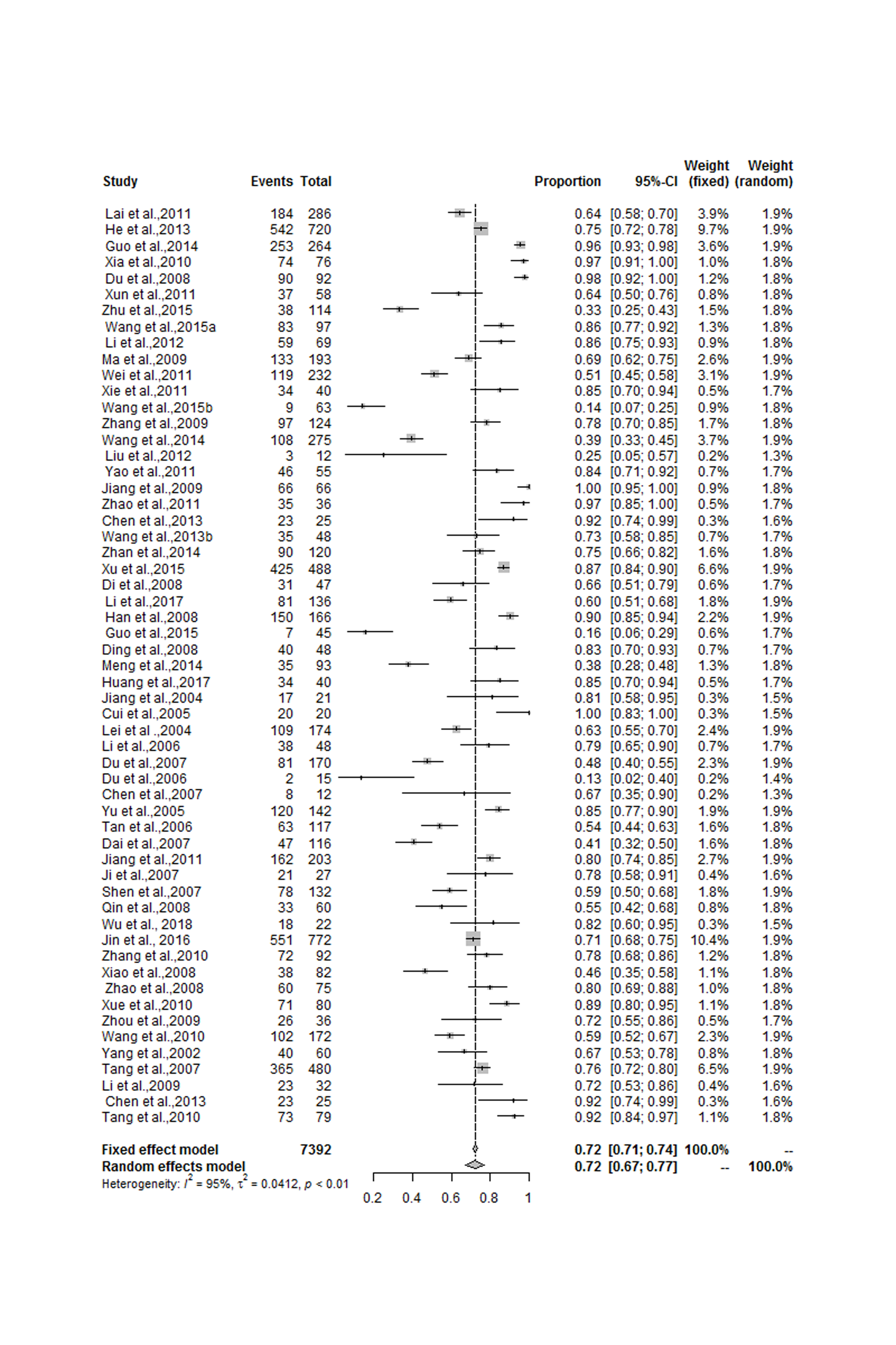

Supplement: S1 Fig — (TIF) [file pone.0228388.s003.tif]

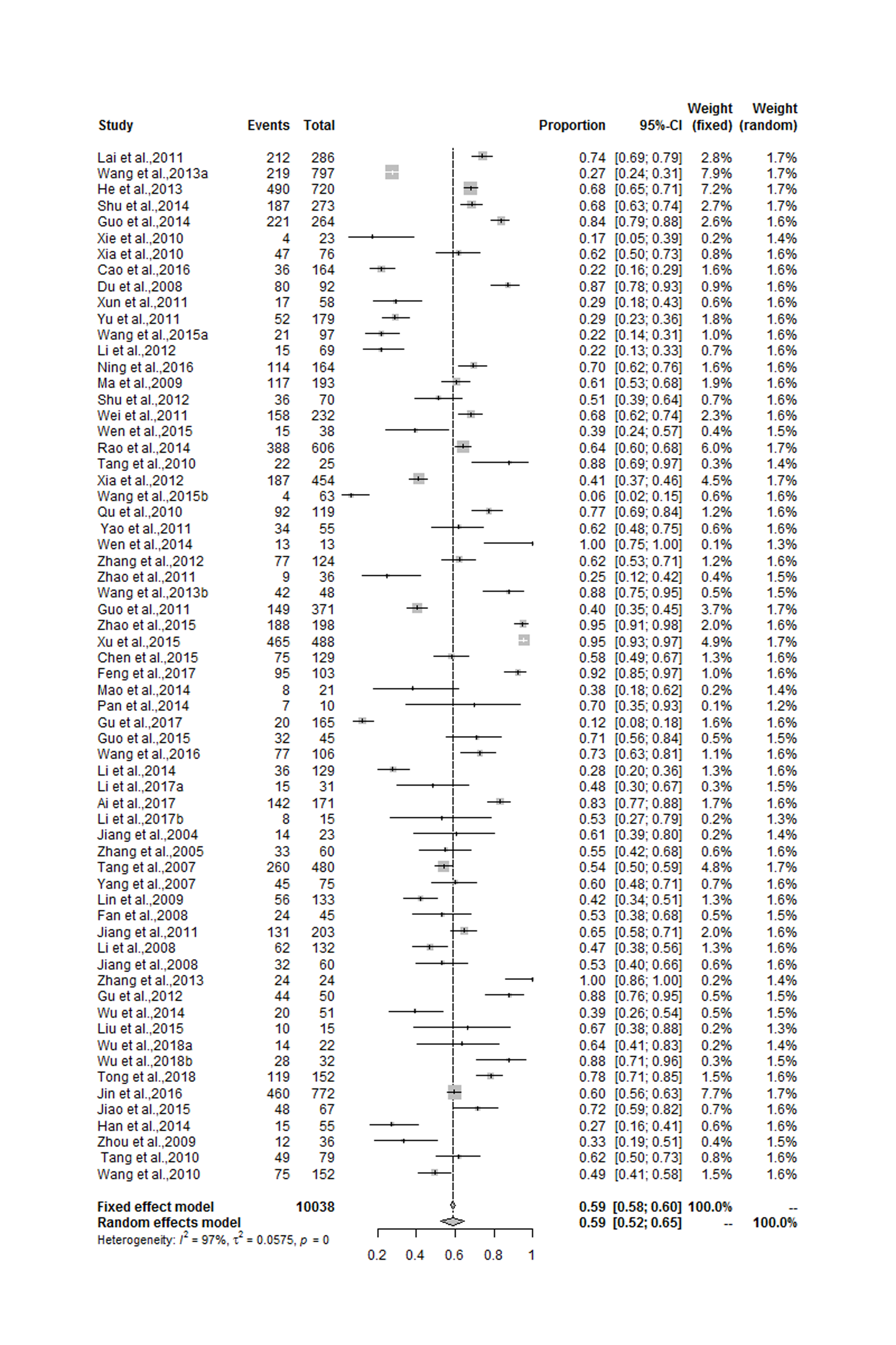

Supplement: S2 Fig — (TIF) [file pone.0228388.s004.tif]
